# Relationships between Obstructive Sleep Apnea Syndrome, Continuous Positive Airway Pressure Treatment, and Inflammatory Cytokines

**DOI:** 10.1155/2014/518920

**Published:** 2014-05-06

**Authors:** Filiz Ünüvar Doğan, Şebnem Yosunkaya, Hacer Kuzu Okur, Ümmügülsüm Can

**Affiliations:** ^1^Department of Chest Diseases, Seydişehir State Hospital, 42370 Konya, Turkey; ^2^Department of Chest Diseases, Meram Medical Faculty, Necmettin Erbakan University, 42090 Konya, Turkey; ^3^Department of Chest Diseases, Fatih Sultan Mehmet Education and Reserch Hospital, 34726 Istanbul, Turkey; ^4^Department of Biochemistry, Konya Education and Research Hospital, 42090 Konya, Turkey

## Abstract

The cardiovascular complications that frequently accompany obstructive sleep apnea syndrome (OSAS) are thought to develop as a result of inflammatory stress associated with cytokines such as IL-6 and TNF-**α**. We conducted the current study to compare levels of these cytokines in OSAS patients (*n* = 33) and nonapneic controls (*n* = 24). Furthermore, we investigated the impact of a three-month regime of continuous positive airway pressure (CPAP) on serum levels of IL-6 and TNF-**α** only in the OSAS patients. There were no significant differences in serum levels of either IL-6 (*P* = 0.782) or TNF- **α** (*P* = 0.722) or TNF-**α** (*P* = 0.722) between OSAS patients and nonapneic controls. Serum IL-6 levels correlated significantly with neck circumference in OSAS patients (*P* = 0.006). In OSAS patients, reduced levels of TNF-**α** and IL-6 correlated with increases in mean SaO_2_ after CPAP treatment (*P* = 0.020 and *P* = 0.051, resp.). However, neither of cytokine levels was significantly impacted by CPAP therapy (both *P* > 0.137). We have demonstrated that plasma cytokine levels are similar in both otherwise healthy subjects with OSAS and in nonapneic control, and we conclude that OSAS-related parameters and CPAP treatment do not play a significant role in altering cytokine levels.

## 1. Introduction

OSAS is a common condition with a number of adverse consequences [1]. The disorder is known to be a major independent risk factor for cardiovascular disease, including both coronary artery disease and cerebrovascular events [2]. During sleep, individuals with OSAS have repeated episodes of declines or cessation of breathing (hypopneas or apneas) due to upper airway obstructions. These obstructions result in the following: excessive daytime sleepiness (EDS), due to interruption of sleep with frequent respiratory arousals; chronic intermittent hypoxia (CIH), due to repetitive decreases in oxygen saturation with rapid reoxygenation; and repeated changes in sympathetic tonus due to apnea-hypopneas [1]. The mechanism by which OSAS is associated with cardiovascular disease is not fully understood, but activation of the inflammatory cascade, potentially involving TNF-*α*, IL-6, or C-reactive protein (CRP), may be an underlying cause of these disease states [3–9]. There is evidence from large epidemiological studies that levels of CRP, IL-6, and TNF-*α* are independent predictors of future cardiovascular events and mortality in subjects with and without known cardiovascular disease [10].

Although the exact mechanism of OSAS-induced increased levels of CRP, IL-6, and TNF-*α* is not known, both sleep deprivation and hypoxemia are believed to be important causative factors [3, 4, 11]. Levels of these inflammatory markers tend to be lower in OSAS patients treated with continuous positive airway pressure (CPAP), a standard therapy that eliminates obstructive events and prevents daytime somnolence and cardiovascular complications [12, 13]. However, there are a handful of studies showing that CPAP therapy does not alter the levels of inflammatory markers [14–17]. Thus, it is still unclear. We evaluated whether repeated apneas, excessive daytime sleepiness (EDS), or hypoxia influenced the levels of inflammatory cytokines in OSAS patients. We also evaluated whether a 3-month CPAP treatment reduced these cytokines levels in patients.

## 2. Material and Methods

### 2.1. Subjects

The following study protocols were approved by our faculty research ethics committee. Participation in the study was voluntary, and all subjects provided written informed consent. Patients were recruited prospectively from a group of individuals who attended our sleep unit for the investigation of possible OSAS. Male subjects recruited for the study were either overweight or obese (BMI > 25 kg/m^2^) [18] and had not previously been diagnosed with or treated for OSAS.

Patients were asked to provide a detailed medical history and fill out a questionnaire about sleep symptoms and EDS (assessed by the Epworth sleepiness scale (ESS)). Patients underwent a basic medical examination for the following: weight, height, blood pressure, and waist and neck circumferences (measured at the level of the umbilicus and the cricoid, resp.). Comorbid disorders were identified by lung function tests, chest radiography, and electrocardiography.

Patients with any of the following characteristics were excluded from the study: history of cerebrovascular or cardiovascular disease, diabetes (previous diagnosis or fasting blood glucose >126 mg/dL), moderate-to-severe hypertension (previous diagnosis or blood pressure >140/90 mmHg), chronic inflammatory diseases, currently taking lipid-lowering medications; age < 18 or > 70 years; a diagnosis of central sleep apnea or Cheyne-Stokes respiration; clinical manifestation of severe chronic obstructive pulmonary disease (COPD) or asthma (postbronchodilator FEV1 <70% predicted). All subjects with apnea-hypopnea index (AHI) <5 were defined as controls (*n* = 24); those with AHI ≥ 15 were diagnosed as having moderate-to-severe OSAS (*n* = 33) and were eligible for CPAP treatment [19].

### 2.2. Sleep Study

Overnight polysomnography (PSG) monitoring was performed on all participants using the VIASYS SleepScreen System (VIASYS Healthcare, Inc., Chicago, IL, USA) and included the following variables: electroencephalogram (4 channels: C3/A2-, C4/A1-, O1/A2, and O2/A1); electrooculogram (2 channels: right, left); electromyogram of submental muscles (3 channels); electromyogram of the anterior tibialis muscle of both legs (2 channels); electrocardiogram; and airflow (assessed with an oronasal cannula and a thermistor). Chest and abdominal efforts (2 channels) were measured using thoracic and abdominal strain gauges, and arterial oxyhemoglobin saturation (SaO_2_: 1 channel) was measured with a finger probe by pulse oximetry.

Sleep staging and respiratory event scoring were performed manually by a single doctor according to the guidelines of the American Academy of Sleep Medicine [19]. Apnea was defined as episodes of airflow cessation lasting ≥10 s, while hypopnea was defined as episodes lasting ≥10 s, airflow limitation of at least 50% accompanying arousal, and/or ≥ 3% desaturation. The AHI was calculated based on the number of apnea and hypopnea episodes per hour of sleep.

During PSG the following variables were recorded: sleep efficiency (SE), calculated as the total sleep time multiplied by time spent in bed; minimum oxygen saturation, the lowest oxygen saturation recorded during sleep; mean oxygen saturation (mean SaO_2_), the average oxygen saturation recorded during sleep; and time SaO_2_ ≥ 90%  (SaO_2_ ≥ 90), defined as the amount of sleep time spent at ≥90% of the saturation level.

### 2.3. CPAP Treatment

Automatic titration of the CPAP pressure (AutoSet Spirit, ResMed Corp., San Diego, CA, USA) was performed in the sleep laboratory under polysomnographic control while patients were being monitored by a trained sleep laboratory technician. The optimum pressure required to prevent the majority of apneas, hypopneas (AHI < 10 events/h), and snoring was confirmed for each patient; this was usually the 95th percentile of pressure. After titration, each subject received a CPAP machine and related accessories and received instructions to use the machine for 3 months. Hour-meter readings were used to assess patient compliance with the CPAP treatment regime. Daily duration of CPAP use was 5.5 ± 0.7 h (mean ± SD). The majority of our patients met the criteria required to consider them “regular” CPAP users. Our final patient sample size was *n* = 27; six individuals were excluded because of poor CPAP compliance (*n* = 3), refusal to undergo re-PSG at the end of the study (*n* = 1), and failure to follow-up (*n* = 2).

At the end of the three-month treatment period, overnight PSG monitoring was performed on patients who use regular CPAP (*n* = 27) using the VIASYS SleepScreen System and blood samples were collected again at the end of the PSG recording between 07:00 and 08:00 a.m.

### 2.4. Cytokine Assays

Blood samples from patients and controls were collected at the end of the PSG recording between 07:00 and 08:00 a.m. Samples were centrifuged at 3000 g for 10 min at 4°C within 1 h of collection. Serum was stored at −80°C prior to further use, and all samples were processed in the same manner.

IL-6 and TNF-*α* levels were measured by enzyme-linked immunosorbent assays (ELISAs) using commercially available human IL-6 (hIL-6) and TNF-*α* (hTNF-*α*) kits (Bender MedSystems GmbH, Vienna, Austria).

### 2.5. Statistical Analysis

Statistical analyses were performed using SPSS version 13.0 (SPSS Inc., Chicago, IL, USA). Parametric variables were expressed as mean ± SD. We used independent *t*-tests to investigate differences between patient (apneic) (*n* = 33) and control groups (nonapneic) (*n* = 24) and paired *t*-tests to assess within-patient changes resulting from CPAP treatment (*n* = 27). Pearson correlations and regression analyses were used to investigate any associations between changes in circulating cytokine levels and CPAP treatment-induced changes in physiological and sleep parameters. Significance was defined as *P* < 0.05.

## 3. Results

General baseline characteristics of the study group are shown in Table 1. We found no significant differences in neck circumference (*P* = 0.090), BMI (*P* = 0.065), sleep efficiency (*P* = 0.243), or blood pressure (*P* > 0.15), between the OSAS patients and the nonapneic control group. Likewise, both groups had similar total cholesterol, TG, HDL, and LDL levels (all *P* > 0.672; data not shown). OSAS patients had significantly larger waist circumferences than control individuals (114.8 ± 9.5 versus 105.1 ± 5.7 cm; *P* < 0.001).

There were no significant differences in serum levels of either IL-6 (OSAS: 0.060 ± 0.011 pg/mL; control: 0.059 ± 0.013 pg/mL; *P* = 0.782) or TNF-*α* (OSAS: 0.128 ± 0.150 pg/mL; control: 0.108 ± 0.027 pg/mL; *P* = 0.722). In OSAS patients only, there was a significant positive correlation between serum IL-6 levels and neck circumference (*P* = 0.006). However, neck circumference was not related to serum TNF-*α* levels in either group (*P* = 0.300). No correlation between IL-6 or TNF-*α* and either AHI, waist circumference, percentage of time with SaO_2_ ≥ 90%, mean SaO_2_, minimum SaO_2_, REM%, NREM3%, or ESS in either the OSAS or control group was observed (*P* > 0.05; Table 2).

### 3.1. Results of CPAP Treatment

The three-month CPAP therapy had no significant effects on BMI, waist circumference, or neck circumference (*P* > 0.137; Table 3). However, following therapy, patients exhibited significantly lower ESS (*P* < 0.01) and experienced significant improvements in AHI (*P* < 0.001), mean SaO_2 _(*P* = 0.042), and *t* ≥ 90 SaO_2_ (*P* < 0.001). Serum IL-6 and TNF-*α* levels were lower following CPAP treatment, but this did not reach significance (*P* = 0.137).

We used regression analysis to assess whether there was any association between changes in either circulating IL-6 or TNF-*α* and CPAP treatment-induced changes in sleep and physiological parameters. The strongest predictor of a change in circulating levels of TNF-*α* and IL-6 levels following CPAP treatment was the change in mean SaO_2_ (*P* = 0.047; Table 4 and *P* = 0.051; Table 5).

## 4. Discussion

We have demonstrated that OSAS patients and nonapneic controls have similar serum levels of IL-6 and TNF-*α*. Further, we found that three months of active CPAP treatment had no effect on the levels of these cytokines, although it did lead to improvements in PSG parameters and subjective sleepiness.

Previous studies, investigating the potential links between these cytokines, OSAS, and CPAP therapy, have reported conflicting data [3–9, 12–17]. These contradictory results may stem from the fact that cytokine levels are influenced by a multitude of factors, such as cardiovascular disease, type 2 diabetes, asthma, hypertension, smoking, obesity, and some medications [2] and the heterogeneity of the study groups. In accordance with our results, Imagawa et al. [15] found similar levels of IL-6 and TNF-*α* in 112 subjects with severe OSAS, compared with 45 BMI-matched controls, and called for close matching of potential confounders. The key pathogenetic mechanisms resulting from OSA, oxidative stress, inflammation, and sympathetic activation also occur in obesity [20]. We took great care to match groups in BMI to establish a role of OSAS in predicting levels of IL-6 and TNF-*α*. Thus, the range in BMI throughout our cohort was small, which might explain why we did not observe any relationship between cytokines and either BMI or waist circumference. We did, however, observe a significant positive correlation between neck circumference and serum IL-6 levels. These observations are in accordance with data published by Carpegneo and colleagues, who found a positive correlation between IL-6 and 8-isoprostane levels in the exhaled breath condensate of OSAS patients and neck circumference [9]. Together, these results suggest that sleep-related upper airways inflammation may play a role in the pathogenesis and natural history of OSAS.

Interestingly, among OSAS patients treated with three months of CPAP, we did observe correlations between mean SaO_2_ and reductions in serum TNF-*α* and IL-6. Therefore, the present results suggest that apnea-related hypoxia is an important determinant of elevated IL-6 and TNF-*α* in patients with OSAS. Previous studies have reported that the percentage of time with SaO_2_ ≥ 90%  (i.e., percentage of total sleep time) was the strongest predictor of monocyte-derived TNF-*α* [4], implying that apnea-related hypoxia activates monocytes in OSAS patients. Ryan et al. [3] reported that the desaturation index was the strongest predictor for elevated TNF-*α* and IL-8 levels in OSAS patients. However, in patients with OSAS, we found that levels of TNF-*α* and IL-6 prior to treatment did not differ significantly from the levels observed posttreatment. It is possible that we did not see a significant difference in cytokine levels owing to our small sample size or the short treatment time. Our data is in accordance with the randomized controlled trial of Kohler and colleagues [17], who found no change in either IL-6 or IFN*γ* after 4 weeks of CPAP therapy. Further, our results are in agreement with Ryan et al.'s [3] uncontrolled study, which found no change in IL-6 levels after a 6-week withdrawal from CPAP.

EDS, the cardinal symptom of OSAS, may be mediated by cytokines, and EDS patients with sleep disorders such as OSAS, narcolepsy, and idiopathic hypersomnia often have high levels of IL-6 and TNF-*α* cytokines [11]. Further, administration of a TNF-*α* antagonist decreases sleepiness in OSAS patients [21]. Despite these recognized patterns, we found no correlation between either TNF-*α* or IL-6 levels and REM%, NREM3%, or ESS in OSAS patients. In fact, we found similar ESS scores in both OSAS patients and nonapneic controls, which may explain the similarities we observed in cytokine levels in the two groups. It is also important to note that daytime sleepiness can be induced not only by reduced or fragmented sleep, as occurring in OSAS, but also by age, circadian factors, and other medical conditions such as obesity [22].

We found that ESS after CPAP was 8.9 ± 5.7 suggesting that some patients had residual hypersomnolence, whereas in most studies subjective sleepiness did not change. OSAS is a chronic, progressive disorder, and it can be present for years before it is diagnosed. Residual sleepiness in OSAS has been described, as well as persistent sleepiness, even in patients on effective CPAP therapy [2]. Our data did not allow us to determine if the sleepiness was a cause of elevated cytokine levels and is a limitation of this study. Another limitation of this study is that we did not use a randomized, placebo-controlled design. This was not possible, as it is considered unethical to withhold CPAP treatment from OSAS patients who require this treatment [7].

## 5. Conclusions

In conclusion, we found no evidence that cytokine levels are associated with OSAS or could be influenced by CPAP treatment. However, we cannot exclude the possibility that CPAP treatment over several months might gradually reduce systemic inflammation, and this should be addressed in future longer term, randomized controlled trials. This is, in fact, suggested by our data showing reduced serum TNF-*α* and IL-6 levels correlated with mean SaO_2_ after CPAP treatment in OSAS patients. In addition we propose that further work is necessary to explore the potential significance of central obesity on the levels of IL-6.

## Figures and Tables

**Table 1:** Characteristics of OSAS patients and nonapneic control individuals.

	OSAS patients *n* = 33	Control individuals *n* = 24	*P* values
Age (years)	45.3 ± 8.5	40.5 ± 9.5	0.110
NC (cm)	44.6 ± 2.9	43.3 ± 3.0	0.090
WC (cm)	114.8 ± 9.5	105.1 ± 5.7	**<0.001**
BMI (kg/m^2^)	31.0 ± 1.7	30.7 ± 1.5	0.065
AHI (events/h)	47.2 ± 23.2	3.6 ± 1.8	**<0.001**
Minimum SaO_2_ (%)	72.8 ± 10.3	85.5 ± 4.5	**<0.001**
Mean SaO_2_ (%)	88.4 ± 2.9	92.9 ± 2.2	**<0.001**
SaO_2_ ≥ 90 (% TST)	47.6 ± 29.2	91.9 ± 12.2	**<0.001**
SE	81.0 ± 9.4	83.8 ± 8.3	0.243
ESS	10.3 ± 5.6	7.6 ± 5.6	0.075
BP systolic	124 ± 17	119 ± 13	0.150
BP diastolic	75 ± 13	72 ± 10	0.341

AWC: waist circumference; NC: neck circumference; BMI: body mass index; AHI: apnea-hypopnea index; Minimum SaO_2_: the lowest oxygen saturation recorded during sleep; mean SaO_2_: the average oxygen saturation recorded during sleep; SaO_2_ ≥ 90 (% TST): the amount of total sleep time spent at ≥90% saturation; SE: sleep efficiency; ESS: Epworth sleepiness scale; BP: blood pressure.

**Table 2:** Results from correlations between physiological/sleep parameters and both IL-6 and TNF-*α* levels in OSAS patients.

	IL-6		TNF-*α*	
	*r*	*P*	*r*	*P*
WC (cm)	0.307	0.082	0.170	0.926
NC (cm)	0.469	**0.006**	0.183	0.300
BMI (kg/m^2^)	0.0184	0.305	0.050	0.784
AHI (events/h)	0.194	0.280	0.189	0.293
NREM3 (%)	0.016	0.928	−0.189	0.291
REM (%)	0.077	0.671	−1.64	0.362
Minimum SaO_2_	0.102	0.572	−2.37	0.184
Mean SaO_2_	0.272	0.126	0.265	0.136
SaO_2_ ≥ 90 (% TST)	0.285	0.108	0.198	0.270
ESS	0.189	0.269	0.034	0.849

AWC: waist circumference; NC: neck circumference; BMI: body mass index; AHI: apnea-hypopnea index; Minimum SaO_2_: the lowest oxygen saturation recorded during sleep; mean SaO_2_: the average oxygen saturation recorded during sleep; SaO_2_ ≥ 90 (% TST): the amount of total sleep time spent at ≥90% saturation; ESS: Epworth sleepiness scale.

**Table 3:** Characteristics of OSAS patients before and after three months of CPAP treatment.

	Pretreatment	Posttreatment	*P* values
TNF-*α* (pg/mL)	0.131 ± 0.171	0.081 ± 0.036	0.279
IL-6 (pg/mL)	0.062 ± 0.077	0.060 ± 0.013	0.137
Minimum SaO_2_ (%)	71.9 ± 10.3	87.7 ± 5.7	0.137
Mean SaO_2_ (%)	88.2 ± 3.13	94.1 ± 1.7	**0.042**
SaO_2_ ≥ 90 (% TST)	47.7 ± 29.9	96.3 ± 6.6	**<0.001**
REM (%)	11.1 ± 6.8	20.4 ± 8.4	**<0.001**
NREM3 (%)	5.0 ± 5.2	7.4 ± 8.1	**<0.001**
ESS	10.9 ± 5.8	8.9 ± 5.7	**<0.001**
AHI (events/h)	43.7 ± 21.8	3.0 ± 3.0	**<0.001**
BMI (kg/m^2^)	33 ± 4	32 ± 3.5	0.137
WC (cm)	114.8 ± 5.3	112.9 ± 6.1	0.182
NC (cm)	44.6 ± 2.9	44.3 ± 3.0	0.201

AWC: waist circumference; NC: neck circumference; BMI: body mass index; AHI: apnea-hypopnea index; Minimum SaO_2_: the lowest oxygen saturation recorded during sleep; mean SaO_2_: the average oxygen saturation recorded during sleep; SaO_2_ ≥ 90 (% TST): the amount of total sleep time spent at ≥90% saturation; ESS: Epworth sleepiness scale.

**Table 4:** Results from correlations and regressions exploring relationships between serum TNF-*α* levels and polysomnographic parameters impacted by CPAP treatment.

	Correlation		Regression	
	*r*	*P*	*t*	*P*
ΔMinimum SaO_2_	−0.271	0.191	−0.144	0.887
ΔMean SaO_2_	−**0.462**	**0.020**	−**2.138**	**0.047**
ΔSaO_2_ ≥ 90 (% TST)	−0.224	0.283	1.372	0.188
ΔESS	0.050	0.811	0.504	0.621
ΔAHI	0.252	0.224	0.099	0.922
ΔREM	−0.087	0.679	−0.284	0.780
ΔNREM	−0.115	0.584	−0.288	0.777

Minimum SaO_2_: the lowest oxygen saturation recorded during sleep; mean SaO_2_: the average oxygen saturation recorded during sleep; SaO_2_ ≥ 90 (% TST): the amount of total sleep time spent at ≥90% saturation; ESS: Epworth sleepiness scale; AHI: apnea-hypopnea index.

**Table 5:** Results from correlations and regressions exploring relationships between serum IL-6 levels and polysomnographic parameters impacted by CPAP treatment.

	Correlation		Regression	
	*r*	*P*	*t*	*P*
ΔMinimum SaO_2_	−0.220	0.290	0.165	0.871
ΔMean SaO_2_	−0.394	**0.051**	1.305	0.209
SaO_2_ ≥ 90 (% TST)	0.268	0.196	0.535	0.600
ΔESS	0.237	0.253	1.289	0.215
ΔAHI	−0.005	0.982	1.333	0.200
ΔREM	−0.057	0.787	−0.716	0.484
ΔNREM	−0.094	0.655	−0.436	0.668

Minimum SaO_2_: the lowest oxygen saturation recorded during sleep; mean SaO_2_: the average oxygen saturation recorded during sleep; SaO_2_ ≥ 90 (% TST): the amount of total sleep time spent at ≥90% saturation; ESS: Epworth sleepiness scale; AHI: apnea-hypopnea index.
